# Targeting tachykinin receptors in neuroblastoma

**DOI:** 10.18632/oncotarget.13440

**Published:** 2016-11-18

**Authors:** Anton G. Henssen, Andrea Odersky, Annabell Szymansky, Marleen Seiler, Kristina Althoff, Anneleen Beckers, Frank Speleman, Simon Schäfers, Katleen De Preter, Kathy Astrahanseff, Joachim Struck, Alexander Schramm, Angelika Eggert, Andreas Bergmann, Johannes H. Schulte

**Affiliations:** ^1^ Molecular Pharmacology Program, Sloan Kettering Institute, Memorial Sloan Kettering Cancer Center, New York, USA; ^2^ Department of Pediatric Oncology and Hematology, University Children's Hospital Essen, Germany; ^3^ Department of Pediatric Oncology/Hematology, Charité- Universitätsmedizin Berlin, Germany; ^4^ Sphingotec GmbH, Hennigsdorf, Germany; ^5^ Center of Medical Genetics Ghent (CMGG), Ghent University Hospital, Belgium; ^6^ German Consortium for Translational Cancer Research (DKTK), Partner Site Charite Berlin, Berlin, Germany

**Keywords:** fosaprepitant, aprepitant, neuroblastoma, NK1R, targeted therapy

## Abstract

Neuroblastoma is the most common extracranial tumor in children. Despite aggressive multimodal treatment, high-risk neuroblastoma remains a clinical challenge with survival rates below 50%. Adding targeted drugs to first-line therapy regimens is a promising approach to improve survival in these patients. TACR1 activation by substance P has been reported to be mitogenic in cancer cell lines. Tachykinin receptor (TACR1) antagonists are approved for clinical use as an antiemetic remedy since 2003. Tachykinin receptor inhibition has recently been shown to effectively reduce growth of several tumor types. Here, we report that neuroblastoma cell lines express *TACR1*, and that targeting TACR1 activity significantly reduced cell viability and induced apoptosis in neuroblastoma cell lines. Gene expression profiling revealed that TACR1 inhibition repressed E2F2 and induced TP53 signaling. Treating mice harboring established neuroblastoma xenograft tumors with Aprepitant also significantly reduced tumor burden. Thus, we provide evidence that the targeted inhibition of tachykinin receptor signaling shows therapeutic efficacy in preclinical models for high-risk neuroblastoma.

## INTRODUCTION

Neuroblastoma is the most common extracranial solid tumor in children. Polychemotherapy is a well-established part of first-line therapy for patients with metastatic neuroblastomas [1]. However, long-term adverse side effects are a problem for survivors and curative treatment remains unsuccessful with standard first-line treatment in most high-risk cases [2]. Additional therapeutic strategies that lower toxicity and/or improve efficacy of current treatment, such as novel drug combination regimens are desperately needed to improve survival and long-term quality of life during long-term survival in high-risk neuroblastoma patients.

Aprepitant (Emend) is approved by the Food and Drug Administration (FDA) and the European Medicines Agency (EMA) for the treatment of chemotherapy-induced nausea and vomiting and is orally administered [3, 4]. Fosaprepitant (Ivemend), is a water-soluble phosphoryl prodrug of aprepitant, which is intravenously administered [5]. Currently, these drugs are used in clinical practice as antiemetics for neuroblastoma patients receiving emetogenic chemotherapy agents including platinum-based chemotherapeutics [3], and no considerable side effects have been observed. Pharmacokinetic analysis showed that intravenous administration of 115 mg fosaprepitant in patients results in peak concentrations of ~3000 ng/ml blood, equivalent to approximately a 5 μM concentration in circulating blood leading to over 90% TACR1 receptor occupancy by aprepitant. In clinical trials a maximum tolerated dose was not defined and no side effects were observed at single doses up to 1000 mg. Thus, high aprepitant concentrations are tolerated without causing considerable side effects [5].

The tachykinin receptor, TACR1 (formerly NK1R), is a specific G protein-coupled receptor [6, 7] and is expressed in many different cell types that respond to tachykinins in a cell type-specific manner. TACR1 is linked to a variety of physiological and biological processes that include the regulation of neurotransmission, pain, inflammation, cell growth and differentiation [8, 9]. Some recent studies have proposed tachykinin receptor involvement in oncogenesis [10–14]. TACR1 activation by substance P initiates phosphoinositide hydrolysis to mobilize intracellular calcium and active calcium-dependent signaling via kinases, such as the SRC kinase [15, 16]. Aprepitant and fosaprepitant act as antagonists of substance P to selectively antagonize TACR1 signaling [3]. Pharmaceutically blocking tachykinin receptor signaling in colon cancer, breast cancer, hepatoblastoma, osteosarcoma, acute lymphoblastic leukemia and neuroblastoma cancer cell lines was found to inhibit cell growth, and treatment of mice with osteosarcoma xenografts resulted in reduced tumor burden. This previous evidence makes tachykinin receptors a putative target for future anticancer strategies [10, 12, 14].

Although neuroblastoma patients often receive aprepitant during treatment, little is known about what effect this might have on the tumor itself. Encouraged by recent observations of antitumor effects of tachykinin receptor antagonists in various cancer cell lines, we investigated the role of TACR1 in high-risk neuroblastoma and explored whether targeting TACR1 could be a therapeutic option in this disease.

## RESULTS

### TACR1 and its downstream targets SRC and p-SRC are expressed in a subset of neuroblastoma cell lines

**Figure 1:**
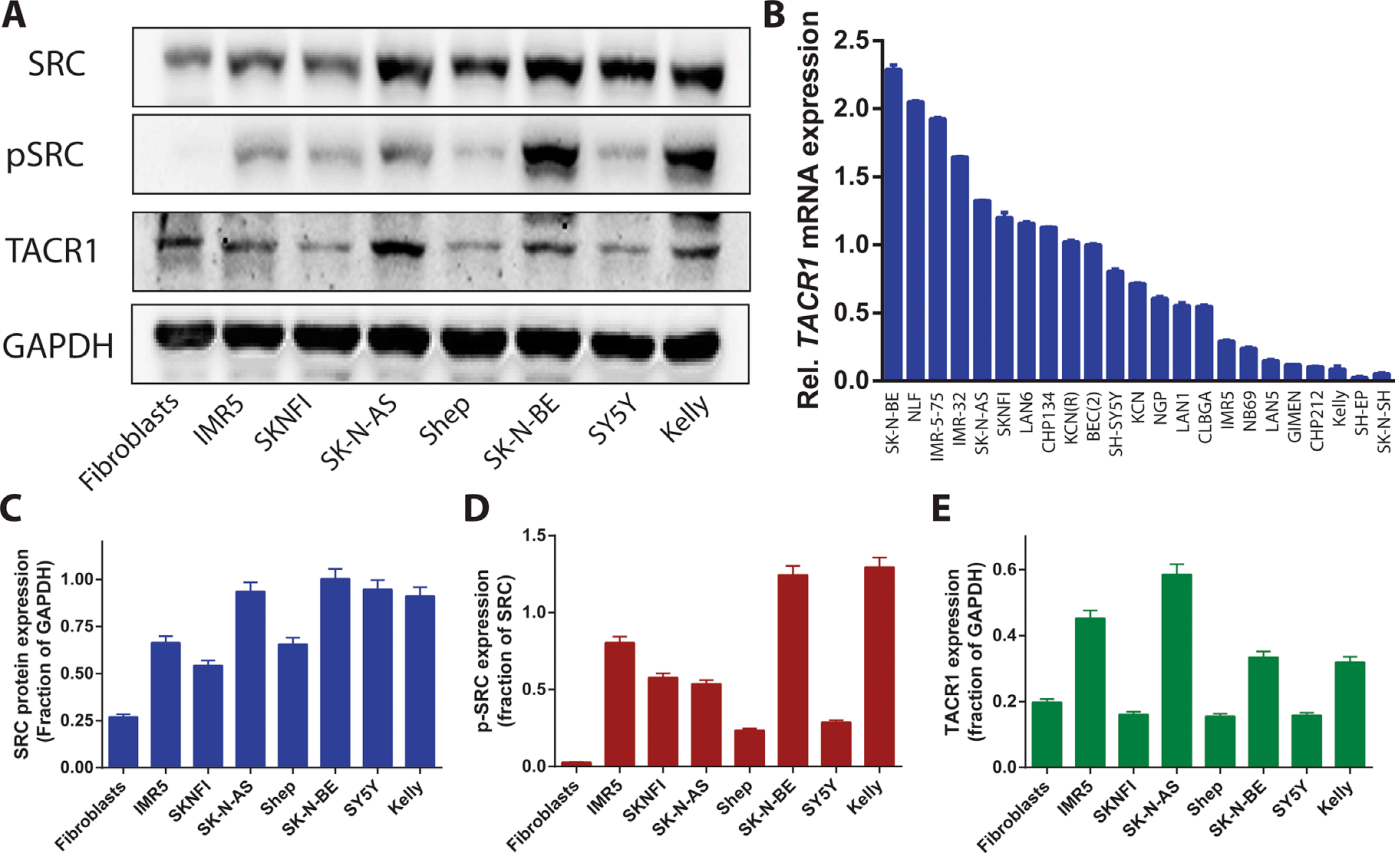
TACR1 and its downstream targets SRC and p-SRC are expressed in neuroblastoma cell lines (**A**) Endogenous TACR1, SRC and p-SRC expression in whole-cell extracts of neuroblastoma cell lines was visualized by western blotting. GAPDH was used as a loading control. (**B**) Quantitative RT-PCR of *TACR1* mRNA in a panel of neuroblastoma cell lines (*n* = 3, error bars indicate standard deviation). (**C**–**E**) Quantification of SRC (C), p-SRC (D) and TACR1 (E) protein expression using densitometry analysis of western immunoblots (*n* = 3, error bars indicate standard deviation).

TACR1 expression was examined in a panel of neuroblastoma cell lines as well as fibroblast control cells using western blotting and quantitative RT-PCR. TACR1 protein was expressed in all human neuroblastoma cell lines assessed, although expression levels varied between cell lines, with SY5Y expressing only low levels and IMR5 strongly expressing TACR1 (Figure 1A, 1B and 1E). Non-transformed fibroblast cells also expressed TACR1 protein (Figure 1A and 1E). To assess TACR1 activity in these cells we measured SRC and phosphorylated SRC (p-SRC) protein expression using western blotting. IMR5, SK-N-BE and Kelly cells expressed the highest levels of p-SRC whereas SY5Y and Shep cells only expressed low levels of p-SRC, indicating different levels of SRC activation in these cells. No measureable p-SRC was detected in non-transformed fibroblasts. Based on the observed TACR1 expression and previous reports of TACR1 mitogenic properties in other tumor entities we hypothesized that TACR1 activity might exhibit mitogenic functions in a subset of neuroblastoma cells through activation of SRC and other downstream targets and that pharmaceutically inhibiting TACR1 could be a therapeutic option in these cells.

### Inhibition of TACR1 signaling inhibits cell growth and leads to increased cell death

**Figure 2:**
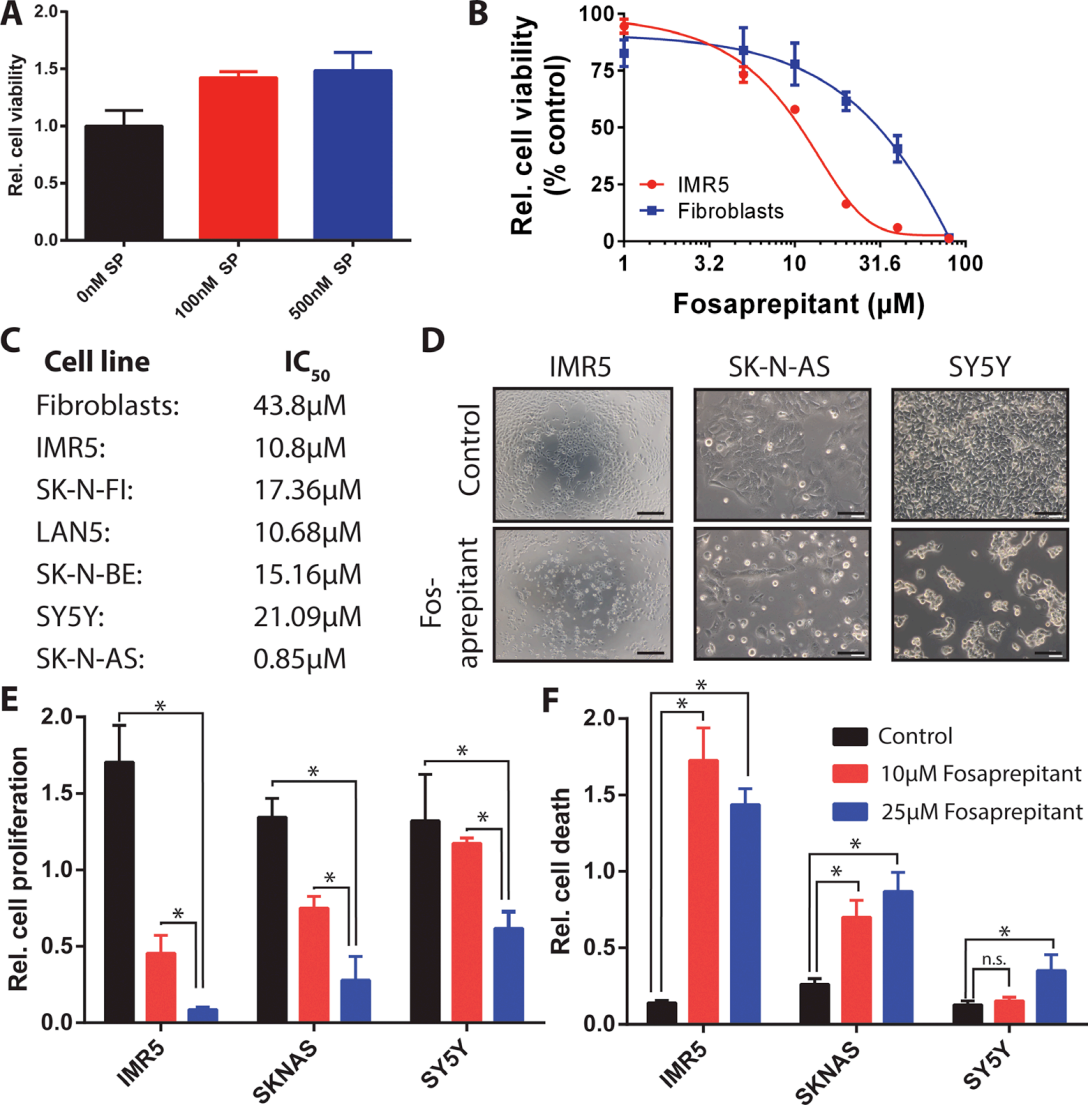
Competitive inhibition of TACR1 with fosaprepitant leads to decreased cell viability and increased cell death in neuroblastoma cell lines (**A**) MTT cell viability assay showing increased number of viable IMR5 neuroblastoma cells after treatment with TACR1 agonist substance P (100 nM and 500 nM, *n* = 3, error bars represent standard deviation, student's *t*-test *p* < 0.05 for 0 nM substance P vs. 100 nM substance P, *p* < 0.05 for 0 nM substance P vs. 500 nM substance P). (**B**) Dose-response of neuroblastoma cell line IMR5 (red) and non-transformed human fibroblasts (blue) after fosaprepitant treatment (*n* = 3, error bars represent standard deviation). (**C**) 50% Inhibitory concentration (IC50) of a panel of neuroblastoma cell lines and non-transformed fibroblast controls measured using MTT assays after treatment with 0–80 μM fosaprepitant. (**D**) Representative images of neuroblastoma cell cultures after 72 h of treatment with 5 μΜ fosaprepitant or Meglumine control (Scale bar = 100 μm). (**E**) BrdU incorporation ELISA assays showing decreased BrdU incorporation in neuroblastoma cell lines treated with fosaprepitant for 72 h in comparison with control-treated cells (*n* = 3, error bars represent standard deviation). (**F**) Cell death ELISA of neuroblastoma cell lines treated with fosaprepitant or control for 72 h, showing induction of cell death following treatment with fosaprepitant (*n* = 3, error bars represent standard deviation). For all figures, asterisk indicates *p* < 0.05 as calculated by student's *t*-test.

To inquire whether TACR1 signaling leads to increased proliferative capacity in neuroblastoma cells, we treated IMR5 cells, which express high levels of TACR1 as well as high levels of p-SRC, with the TACR1 ligand, substance P. Treatment with substance P increased the relative number of viable IMR5 cells over time in 3-(4,5-dimethylthiazol-2-yl)-2,5-diphenyltetrazolium bromide (MTT) assays (Figure 2A), suggesting that TACR1 activity might be required for IMR5 cell survival. To assess the effect of TACR1 inhibition on neuroblastoma cell viability, we treated cells *in vitro* with the water soluble aprepitant analog, fosaprepitant, and assessed cell viability relative to untreated control cultures in MTT assays. All neuroblastoma cell lines tested exhibited a concentration-dependent reduction in cell viability in response to TACR1 inhibition (Figure 2B and 2C), although sensitivity varied strongly among cell lines. While untransformed fibroblasts tolerated high concentrations of fosaprepitant (IC50 = 44 μM), only 0.85 μM fosaprepitant was needed to induce 50% growth inhibition (IC50) in SK-N-AS cells, indicating a specific effect on malignant cells (Figure 2B and 2C). In addition to assessing cell viability, we investigated proliferative capacity directly by measuring BrdU incorporation in 3 cell lines after fosaprepitant treatment in comparison to untreated control cultures. Cells lines were selected that expressed different levels of TACR1 and p-SRC and that spanned the fosaprepitant sensitivity range identified by assessing cell viability, namely SK-N-AS (highly sensitive), IMR5 (intermediately sensitive) and SY5Y (relatively insensitive). BrdU incorporation was significantly suppressed after fosaprepitant treatment (Figure 2E). Interestingly, the most pronounced reduction in BrdU incorporation occurred in IMR5 cells, which express the highest TACR1 and p-SRC levels of the 3 cell lines whereas SY5Y cells expressing low levels of TACR1 and p-SRC showed relative resistance to fosaprepitant treatment. We next assessed whether fosaprepitant treatment also induced cell death by measuring the relative amount of cytoplasmic histone-associated DNA fragments in cells treated with fosaprepitant compared to untreated control cells. Fosaprepitant treatment significantly increased the relative amount of histone-associated DNA in all 3 neuroblastoma cell lines tested, indicating that fosaprepitant induces cell death (Figure 2F). Again, IMR5 cells showed the most pronounced increase in cell death, suggesting that fosaprepitant effects on neuroblastoma cells might be dependent on TACR1 signaling activity.

### Inhibition of TACR1 signaling leads to increased apoptosis and cell cycle arrest depending on the cellular context

**Figure 3:**
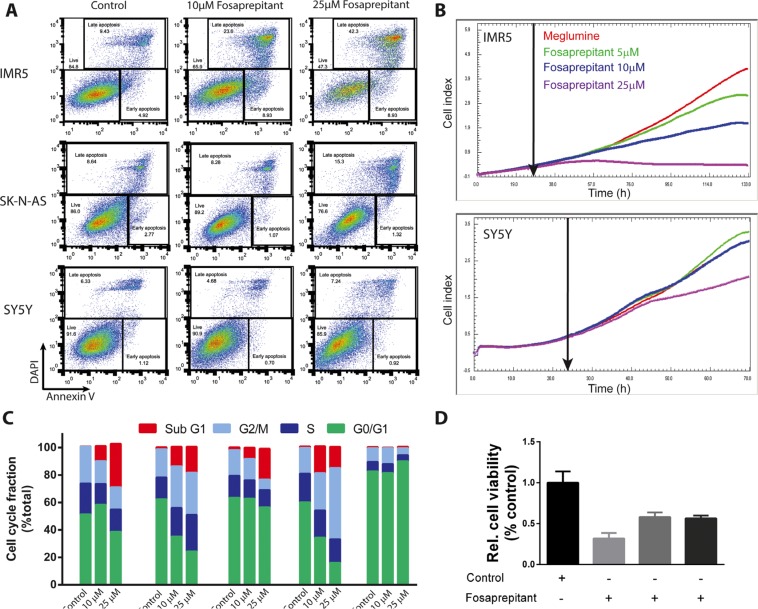
Inhibition of TACR1 with fosaprepitant leads to apoptosis and cell cycle arrest in neuroblastoma cell lines (**A**) Representative flow cytometry images of IMR5, SK-N-AS and SY5Y cells treated with fosaprepitant or control for 72 h and stained for Annexin V and DAPI. Gates show viable cells (DAPI negative, Annexin V negative), early apoptotic cells (DAPI negative, Annexin V positive) and late apoptotic cells (DAPI positive, Annexin V positive) after treatment with fosaprepitant or control. (**B**) Proliferation of cells monitored in real time using the Xcilligance system after fosaprepitant treatment compared with cells treated with Meglumine control (*n* = 3, line represents mean value). (**C**) Fraction of cells in each point of the cell cycle measured after 48 h of treatment with fosaprepitant or meglumine (control). (**D**) Bar graph showing cell viability in MTT assays of IMR5 cells treated with 5 μM fosaprepitant or control for 72 h as well as combined fosaprepitant and substance P treatment for 72 h (+ = 100 nM Substance P, ++ = 500 nM Substance P, *n* = 3, error bars represent the standard deviation)(100nM vs. no substance P, *p* = 0.0013; 500 nM vs. no substance P, *p* = 0.0006).

Considering the differential anti-tumoral effects of fosaprepitant on different neuroblastoma cell lines, we set out to assess the cellular mechanisms responsible for the antitumoral activity of fosaprepitant. To assess whether cells were dying by apoptosis, we flow cytometrically assessed cell surface expression of annexin V in all three cell lines after treatment with fosaprepitant. Fosaprepitant treatment significantly increased both the fraction of apoptotic and pre-apoptotic in IMR5 and SK-N-AS cells but not in SY5Y cells (Figure 3A). To assess whether the differential response of IMR5 and SY5Y cells was due to kinetic differences in cell proliferation, we monitored real-time cell growth using xCelligence [17]. We observed that fosaprepitant led to a decrease in IMR5 cell numbers within 48 h after treatment, consistent with induction of cell death, whereas SY5Y cells grew exponentially with only modestly decreased growth rates at high fosaprepitant doses (Figure 3B). To examine the underlying cellular processes occurring in cells treated with fosaprepitant, we assessed the cell cycle distribution of cells treated with fosaprepitant. Consistent with our observation of increased cell death and apoptosis in IMR5 and SK-N-AS cells, the fraction of IMR5 and SK-N-AS cells in sub-G1 increased after treatment with fosaprepitant (Figure 3C). In SY5Y cells on the other hand, we observed an increase in the fraction of cells in G2M phase, consistent with reduced induction of apoptosis/cell death (Figure 3C). As observed before, only minimal changes in the cell cycle distribution of non-transformed fibroblasts were observed after fosaprepitant treatment. To test whether the effect of fosaprepitant on IMR5 cells were specifically due to TACR1 inhibition, competition experiments with the TACR1 agonist, substance P were carried out. IMR5 cells were incubated with 100 nM or 500 nM substance P 1h before fosaprepitant treatment to saturate cell surface receptors. When incubated with substance P we observed a > 1.8 fold increase in the relative number of viable cells after fosaprepitant treatment (Figure 3D). However, even 500 nM substance P was not able to reverse the effects of fosaprepitant in IMR5 cell lines to the full extent. This is consistent with the previously described higher binding efficiency of fosaprepitant to TACR1 [5, 18]. The partial reversibility of fosaprepitant-induced growth inhibition by nanomolar substance P concentrations as well as the differential activity of fosaprepitant on IMR5 and SY5Y cells suggests selective targeting of TACR1 by fosaprepitant. The antiproliferative effects of fosaprepitant are largely selective for transformed cells, suggesting that off-target effects or general cytotoxicity in treated patients should be low.

### TACR1 inhibition disrupts oncogenic gene expression signatures and induces gene sets associated with apoptosis

**Figure 4:**
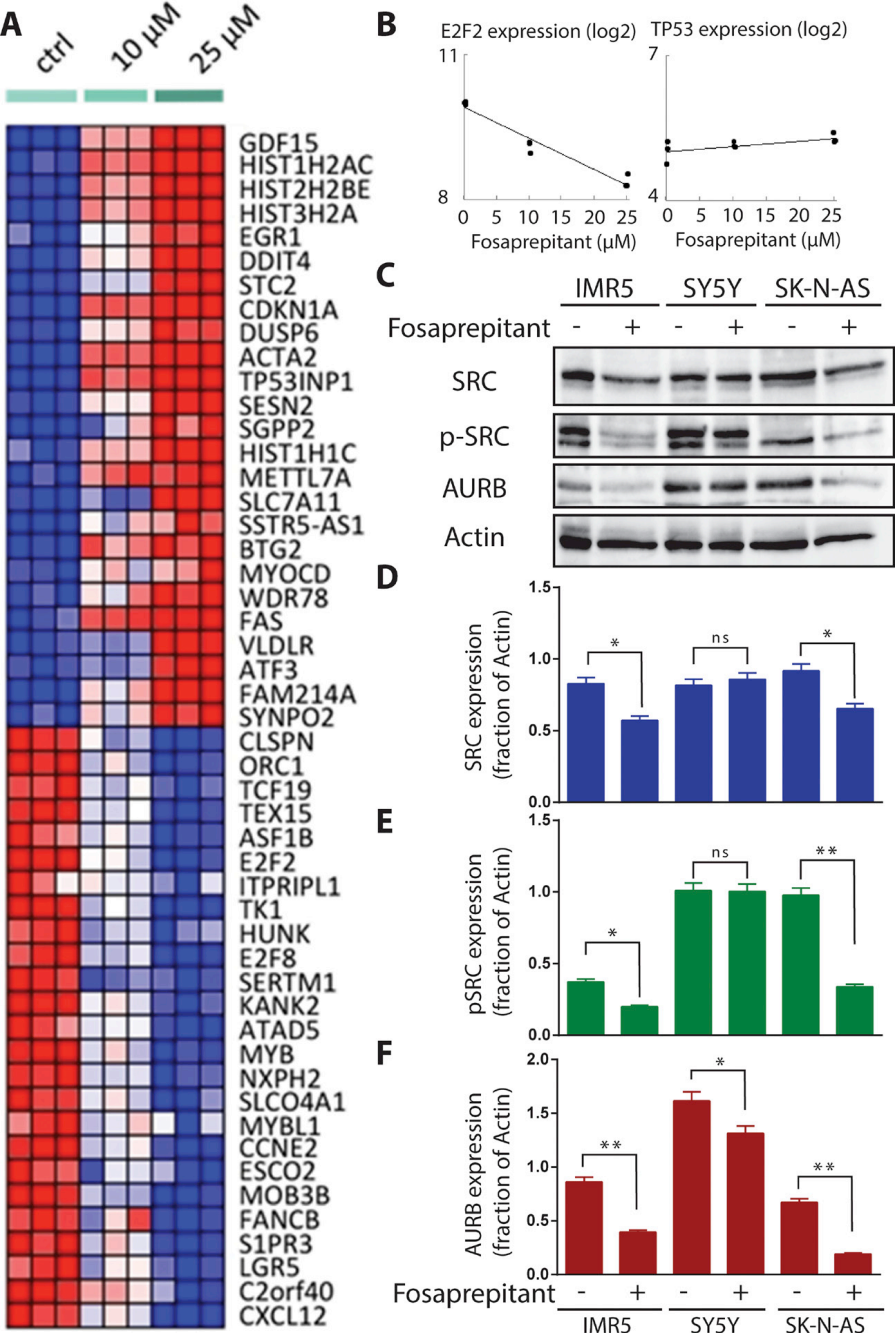
TACR1 inhibition by fosaprepitant leads to decreased SRC phosphorylation and is associated with dose-dependent significant and specific gene expression changes (**A**) Heatmap representation of top 50 dose-sensitive up- (red) and downregulated (blue) genes upon treatment of IMR-5 with fosaprepitant or control (heatmap representation generated by HeatmapViewer of GenePattern servers). (**B**) Dose-dependent mRNA expression changes of *E2F2* and *TP53* after treatment of IMR5 cells with fosaprepitant or control as measured using gene-expression arrays. (**C**) Western blot of published downstream effectors of TACR1 signaling, SRC and p-SRC, as well as the newly discovered potential downstream target AURB after treatment of IMR5, SY5Y and SK-N-AS with fosaprepitant or control. (**D**–**F**) Quantification of SRC (D), p-SRC (E) and TACR1 (F) protein expression using densitometry analysis of western immunoblots (*n* = 3, error bars indicate standard deviation, * indicates *p* < 0.05 and ** indicates *p* < 0.01 as calculated by student's *t*-test).

To gain further insight into the molecular basis for antitumor effects observed *in vitro* from fosaprepitant treatment, we analyzed gene expression in IMR5 cells following treatment with increasing concentrations of fosaprepitant or control. A clear dose-repose was apparent in expression profiles indicating a specific molecular mechanism underlying these expression changes (Figure 4A). Known oncogenes such as *MYB and AURB* were among the top downregulated genes following treatment (Figure 4A). To reveal potential novel downstream targets of TACR1 in neuroblastoma cells and understand the mechanisms through which TACR1 inhibition leads to apoptosis in neuroblastoma cells, we interrogated our gene expression data with published, validated gene signatures to assess statistically significant enrichment by GSEA. Consistent with the phenotypic changes observed in neuroblastoma cells treated with fosaprepitant (Figures 2 and 3), the top 20 upregulated gene sets in IMR5 cells treated with fosaprepitant represented either pathways involved in apoptosis or cell cycle arrest, whereas the top 20 downregulated gene sets were enriched in gene sets involved in proliferation and progression through the cell cycle (Table 1). To identify common downstream effectors of TACR1 signaling, we searched for known regulons in the genes repressed after fosaprepitant treatment, and detected a significant enrichment of motifs bound by the E2F2 transcription factor (Table 2). This suggests E2F2 as a downstream target of TACR1. Consistent with this hypothesis, E2F2 itself was repressed in a dose-dependent manner after fosaprepitant treatment (Figure 4B). Genes upregulated by fosaprepitant were also searched for regulons, and were enriched for TP53-binding motifs (Table 2). *TP53* expression was only slightly increased by fosaprepitant in IMR5 cells (Figure 4D, not significant), suggesting changes in TP53 activity rather than TP53 levels. Our analyses therefore suggests that TACR1 inhibition in neuroblastoma cells represses E2F2 and induces the pro-apoptotic TP53 pathway, indicating that E2F2 and TP53 may be downstream targets of TACR1 signaling. To test whether transcriptional changes translated into significant protein expression changes, we assessed AURB expression in cells treated with fosaprepitant or control. Consistent with the significant reduction in *AURB* mRNA expression, we observed a significant reduction of AURB protein in IMR5 and SK-N-AS cells following fosaprepitant treatment (Figure 4C). Previously published data suggested that SRC signaling is critical for the pro-tumoral activity of TACR1 [16]. We therefore tested the effect of TACR1 inhibition on SRC phosphorylation and expression in IMR5, SY5Y and SK-N-AS cells. P-SRC expression decreased significantly after 48 hours of fosaprepitant treatment in IMR5 and SK-N-AS cells whereas p-SRC did not decrease significantly in SY5Y cells, that in IMR5 and SK-N-AS cells TACR1 signals at least in part through SRC activation/phosphorylation (Figure 4C–4F). Collectively, our results suggest that the antitumor effects induced by fosaprepitant in neuroblastoma cells may be due, at least in part, to decreased SRC phosphorylation leading to downstream E2F2 repression and that apoptosis might be induced via activation of pro-apoptotic TP53 signaling.

**Table 1:** Fosaprepitant treatment leads to significant and specific gene expression changes in TP53, EGFR and chemotherapy induced gene sets

Gene Set	Rank	Size	NES	FDR q-val
*Upregulated*				
ZHANG TLX TARGETS DN	1	66	2.56	0.00
PODAR RESPONSE TO ADAPHOSTIN UP	2	116	2.53	0.00
KERLEY RESPONSE TO CISPLATIN UP	3	29	2.50	0.00
SMIRNOV RESPONSE TO IR 6HR UP	4	133	2.45	0.00
KRIGE AMMINO ACID DEPRIVATION	5	25	2.41	0.00
KANNAN TP53 TARGETS UP	6	47	2.39	0.00
WARTERS RESPONSE TO IR SKIN	7	56	2.36	0.00
ZHANG TLX TARGETS 60HR UP	8	200	2.36	0.00
KOBAYASHI EGFR SIGNALING 24HR UP	9	69	2.33	0.00
CHANG CORE SERUM RESPONSE DN	10	148	2.29	0.00
WARTERS IR RESPONSE 5GY	11	31	2.29	0.00
MOLENAAR TARGETS OF CCND1 AND CDK4 UP	12	52	2.27	0.00
TIEN INTESTINE PROBIOTICS 24HR DN	13	189	2.26	0.00
ODONNELL TFRC TARGETS UP	14	225	2.25	0.00
GHANDHI DIRECT IRRADIATION UP	15	54	2.25	0.00
ZHANG TLX TARGETS 36HR UP	16	155	2.23	0.00
VARELA ZMPSTE24 TARGETS UP	17	29	2.23	0.00
RUIZ TNC TARGETS UP	18	106	2.21	0.00
ONDER CDH1 TARGETS 1 UP	19	92	2.21	0.00
PRAMOONJAGO SOX4 TARGETS UP	20	43	2.21	0.00
*Downregulated*				
DUTERTRE ESTRADIOL RESPONSE 24HR UP	1	283	−3.44	0.00
ROSTY CERVICAL CANCER PROLIFERATION CLUSTER	2	136	−3.21	0.00
KOBAYASHI EGFR SIGNALING 24HR DN	3	223	−3.20	0.00
ZHANG TLX TARGETS 60HR DN	4	254	−3.17	0.00
FUJII YBX1 TARGETS DN	5	188	−3.12	0.00
ZHOU CELL CYCLE GENES IN IR RESPONSE 6HR	6	79	−3.08	0.00
SOTIRIOU BREAST CANCER GRADE 1 VS 3 UP	7	144	−3.06	0.00
SARRIO EPITHELIAL MESENCHYMAL TRANSITION UP	8	149	−3.03	0.00
MANALO HYPOXIA DN	9	272	−3.02	0.00
CROONQUIST IL6 DEPRIVATION DN	10	93	−3.00	0.00
ZHANG TLX TARGETS UP	11	84	−2.99	0.00
KONG E2F3 TARGETS	12	86	−2.99	0.00
BURTON ADIPOGENESIS 3	13	91	−2.98	0.00
BLUM RESPONSE TO SALIRASIB DN	14	320	−2.98	0.00
MOLENAAR TARGETS OF CCND1 AND CDK4 DN	15	54	−2.96	0.00
GRAHAM NORMAL QUIESCENT VS NORMAL DEVIDING DN	16	77	−2.95	0.00
ZHOU CELL CYCLE GENES IN IR RESPONSE 24HR	17	112	−2.93	0.00
CROONQUIST NRAS SIGNALLING DN	18	72	−2.91	0.00
ZHANG TLX TARGETS 36HR DN	19	176	−2.91	0.00
SHEDDEN LUNG CANCER POOR SURVIVAL A6	20	408	−2.90	0.00

Table of top 20 up- and down-regulated gene sets from the MSigDB C2 collection, enriched among genes induced by inhibition of the TACR1 based on GSEA (rank, rank of gene set in overall list of gene sets, ranked according to decreasing NES; size, number of genes in each set; NES, normalized enrichment score).

**Table 2:** Fosaprepitant leads to gene expression changes at a unique subset of transcription factor targets

Transcription factor	NES	#targets	#motifs
***Upregulated genes***			
TP53	5.65	86	6
FOXO4	5.20	117	35
NFYA	5.02	92	12
JDP2	4.40	80	8
MEIS1	3.74	52	4
HOXA13	3.63	44	3
***Downregulated genes***			
E2F2	9.78	112	31
BRF1	3.70	32	2
TBP	3.54	40	5
KLF7	3.26	6	1

Table of transcription factors that bind to the motifs enriched among the genes up- and down-regulated upon administration of a low dose of the TACR1 antagonist to the IMR-5 cells (NES, normalized enrichment score, # targets, the number of merged targets found by numerous motifs, # motifs).

### Fosaprepitant treatment lowers tumor burden in a human neuroblastoma xenograft mouse model

**Figure 5:**
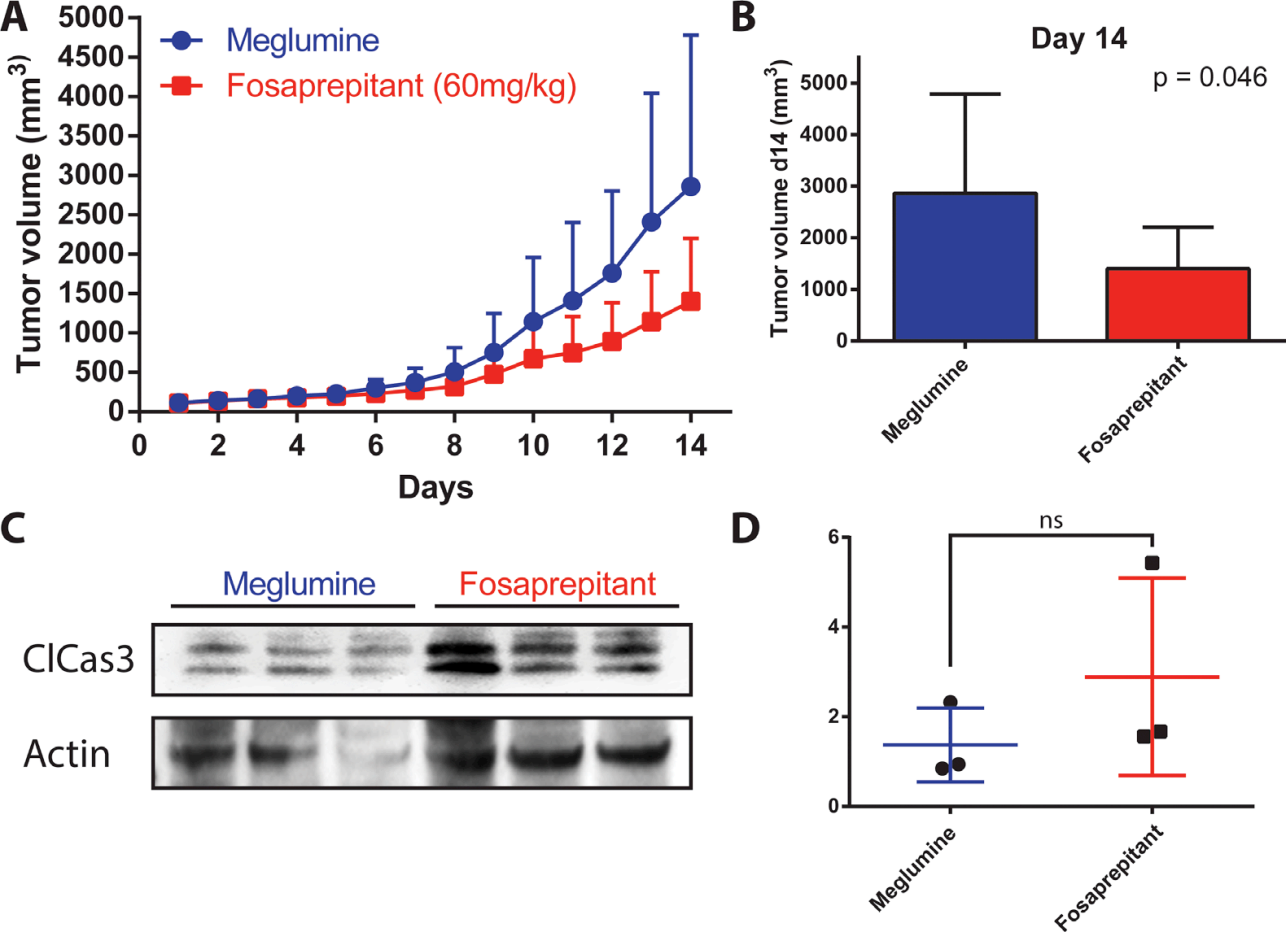
Treatment with fosaprepitant decreases neuroblastoma tumor burden in a xenograft model (**A**) Tumor volumes (mm^3^) of IMR5 xenografts in nude mice after treatment with fosaprepitant (60 mg/kg body weight per day) or control (*n* = 7, error bars represent the standard deviation). (**B**) Tumor volume at day 14 after engraftment of IMR5 xenografts after treatment with fosaprepitant (60 mg/kg body weight per day) compared to control. (**C**) Western blot of cleaved caspase-3 in protein lysates from IMR5 xenografts treated with fosaprepitant (60 mg/kg body weight per day) or control (each column represents an independent biological replicate). (**D**) Quantification of cleaved caspase-3 protein expression using densitometry of western blots from IMR5 xenografts treated with fosaprepitant (60 mg/kg body weight per day) or control (*p* = 0.33 using an unpaired student's *t*-test).

Having observed a robust, selective and specific antitumor effect *in vitro*, we analyzed the effect of TACR1 antagonist fosaprepitant in an *in vivo* model to test whether fosaprepitant could be useful in a clinically relevant context. Xenograft tumors were established subcutaneously from IMR5 cells in nude mice. Mice were randomly separated into control and treatment groups (*n* = 7) and subsequently intraperitoneally injected with 60 mg fosaprepitant/kg body weight/day or with dimeglumine as vehicle control. Consistent with previous reports, fosaprepitant was well tolerated by the mice and produced no significant side effects during the 14 days of treatment. Treatment with fosaprepitant significantly delayed xenograft tumor growth (Figure 5A and 5B) In contrast to our *in vitro* observations, fosaprepitant was not able to stop tumor growth, suggesting reduced *in vivo* pharmacologic activity of fosaprepitant. To test whether fosaprepitant was able to induce apoptosis in IMR5 xenografts, we assessed cleaved caspase-3 expression in three tumors treated with fosaprepitant or vehicle control. Consistent with the decreased anti-tumoral effects *in vivo* compared to *in vitro*, cleaved caspase-3 expression was only slightly increased in tumor treated with fosaprepitant compared to vehicle treated controls (Figure 5C and 5D). Our results support that inhibiting TACR1 with the antagonist, fosaprepitant, provides a therapeutic effect in a mouse model of neuroblastoma, and present fosaprepitant as a possible clinical avenue for patients with neuroblastomas with high-level *TACR1* expression.

### Pretreatment with fosaprepitant synergizes with cytotoxic chemotherapy in neuroblastoma cells

**Figure 6:**
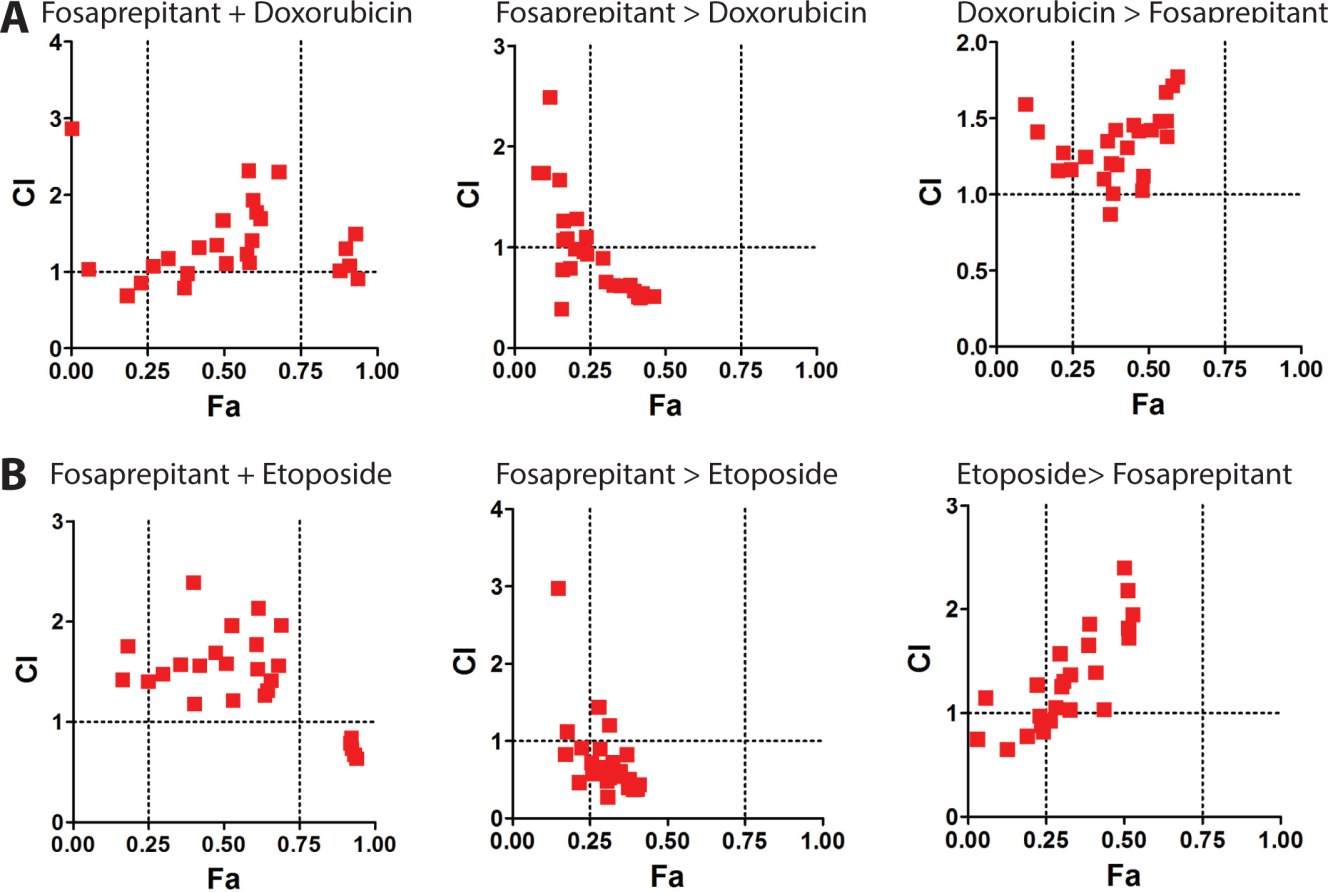
Pretreatment of neuroblastoma cells with fosaprepitant synergizes with cytotoxic chemotherapy A+B Synergy between fosaprepitant and doxorubicin (**A**) and etoposide (**B**) was quantified by Combination Index (CI) analysis vs. fraction affected using CompuSyn (http://www.combosyn.com/). By this method, CI < 1 indicates synergy; CI = 1 indicates an additive effect; and CI > 1 indicates antagonism. Synergy was calculated based on MTT assays of IMR5 cells: i. co-treated with fosaprepitant and doxorubicin (A) or etoposide (B) for 48 hours (left), ii. treated with fosaprepitant for 24 hours followed by 24 hours of doxorubicin (A) or etoposide (B) treatment (middle) or iii. treated with doxorubicin (A) or etoposide (B) for 24 hours followed by fosaprepitant treatment for 24 hours (right).

Considering the non-curative *in vivo* antitumoral activity of fosaprepitant observed (Figure 5), we decided to test whether combination treatment of fosaprepitant with cytotoxic agents could lead to greater anti-tumoral effects in neuroblastoma cells. To test this, we treated IMR5 cells with Doxorubicin and Etoposide, two established chemotherapeutic agents in use for patients with neuroblastoma. We tested co-treatment of IMR5 cells with a chemotherapeutic agent and fosaprepitant for 48 hours as well as pre- or posttreatment of fosaprepitant for 24 hours after/before 24 hours of treatment with a cytotoxic agent. Interestingly, pretreatment of cells with fosaprepitant before both Etoposide and Doxorubicin treatment showed synergistic anti-tumoral effects as evidenced by combinatorial indices (CI) below 1, indicating that fosaprepitant treatment leads to a relative sensitization of neuroblastoma cells to cytotoxic agents (Figure 6A and 6B). Co-treatment as well as post-chemotherapy treatment of cells with fosaprepitant, however, did not increase the antitumoral activity of these agents. These results indicate that scheduling of combination treatment with fosaprepitant and cytotoxic agents has significant effects on their antitumoral activity, which may be of clinical importance for patients receiving these agents.

## DISCUSSION

Here we show that human neuroblastoma cell lines express TACR1 and that blocking TACR1 activity using fosaprepitant robustly inhibits tumorigenic characteristics in both *in vitro* and *in vivo* neuroblastoma models. This is in line with previous reports by Munoz M. et al. describing anti-tumoral activity of fosaprepitant in various adult tumor cell lines [10, 13, 14]. Similar to previous reports, we observed that inhibiting TACR1 with fosaprepitant did not affect all cell lines to the same degree. We observed that fosaprepitant reduced cell viability most strongly in cells expressing high levels of TACR1 and its downstream target p-SRC, i.e. exhibiting high TACR1 activity. Consistent with this, we also observed a more pronounced induction of cell death/apoptosis in cells expressing high levels of TACR1 and p-SRC, whereas cells expressing low levels of TACR1 and p-SRC underwent cell cycle arrest rather than cell death. TACR1 agonist substance P partially restored neuroblastoma cell viability, consistent with on target activity of fosaprepitant and in line with the previously reported stronger binding affinity of fosaprepitant to TACR1 [18]. Based on our data as well as previous reports, we conclude that the antitumoral activity of fosaprepitant is, indeed, due to selective TACR1 inhibition, and that TACR1 expression as well as SRC phosphorylation in primary neuroblastoma samples may be predictive for fosaprepitant sensitivity. Together, our data extends previous knowledge about the mitogenic role of TACR1 in neuroblastoma and establishes TACR1 as a novel therapeutic target for neuroblastoma.

There are only a few reports about the molecular functions of TACR1 and substance P in malignant cells, and little is known about downstream targets in the TACR1 signaling cascade [16, 19, 20]. TACR1 activation has previously been reported to result in phosphoinositide hydrolysis, calcium mobilization and subsequent calcium-dependent signaling activation via kinases such as SRC [6, 7, 16]. We therefore investigated fosaprepitant-induced effects on these previously described targets downstream of TACR1 in neuroblastoma cells. Similar to previous reports in other tumor entities, we detected decreased SRC phosphorylation following TACR1 inhibition, indicating that in neuroblastoma TACR1 might at least in part signal through SRC. Consistent with decreased SRC signaling, expression of *MYB, AURKB* and *PCNA* was repressed in global gene profiles and genes involved in apoptosis (i.e. *FAS*) were upregulated. Global expression profiling also identified *E2F2* and genes highly enriched for E2F2 binding sites to be repressed. The dose dependency of fosaprepitant-induced gene expression changes suggests these are on-target effects. Genes containing TP53 binding motifs were also dose-dependently increased following TACR1 inhibition indicating that TACR1 signaling might directly affect TP53 pathway activity. Our global expression profiling also implicated TACR1 in control of cell cycle progression and apoptosis in neuroblastoma cells, with TACR1 inhibition driving the cell towards apoptosis and cell cycle arrest. This is in line with our observation that cells treated with fosaprepitant undergo apoptosis and cell cycle arrest and is consistent with previous reports showing that TACR1 signaling plays important roles in controlling cell proliferation of various cell types, for review see Datar et al. [8]. It is still not entirely clear how TACR1 signaling affects these pathways. Previous reports and our current data, however, support the direct involvement of TACR1 signaling in regulating the E2F/TP53 signaling in neuroblastoma cells at least in part through activation of SRC.

TACR1 antagonists have been previously described to have *in vivo* antitumoral activity against glioma and breast cancer xenografts [21, 22]. Similarly, we observed that intraperitoneal application of fosaprepitant in mice harboring neuroblastoma xenografts reduced tumor burden. Previous reports together with our current data suggest that fosaprepitant can exert significant therapeutic effects *in vivo*. However, the antitumor effect observed in our model as well as in previous reports was not curative, suggesting that higher fosaprepitant doses may be necessary to more efficiently combat the tumor. Alternatively, combining TACR1 antagonists with other therapeutic agents may also enhance antitumor efficacy. Considerable synergism between fosaprepitant and cytotoxic agents currently used in patients with neuroblastomas has been reported for osteosarcoma cells [13]. Similarly, we observed that fosaprepitant treatment synergized with doxorubicin and etoposide in neuroblastoma cells. Interestingly only pretreatment of cells with fosaprepitant followed by treatment with cytotoxic agents showed considerable synergy, indicating that fosaprepitant might sensitize cells to cytotoxic agents. This suggests that fosaprepitant might prime cells for apoptosis induced by cytotoxic agents, which is consistent with our observation that TP53 signaling was increased by fosaprepitant treatment and that TP53 signaling is required for cytotoxic agent-induced cell death [23–25]. Considering that TP53 as well as AURB were induced by fosaprepitant treatment in our experiments, agents activating or stabilizing TP53 as well as AURB kinase inhibitors may also act synergistically with TACR1 inhibitors in neuroblastoma.

Intravenous administration of 115 mg fosaprepitant in patients results in peak concentrations of 5 μM circulating in blood and doses of up to 1000 mg have been administered to adults without significant side effects [5]. Furthermore, fosaprepitant has been successfully used as an antiemetic in combination with cytotoxic agents such as carboplatin without significant side effects [3]. Based on this, we believe that administering fosaprepitant to patients in parallel with chemotherapeutic agents currently used for first-line therapy of neuroblastoma should be well tolerated. Considering we observed IC_50_ concentrations ranging between 0.85 μM and 21.09 μM for neuroblastoma cell lines *in vitro*, fosaprepitant may have a therapeutic index in patients with neuroblastoma. In summary, the anti-neuroblastoma activity observed in our mouse model and the previously reported good pharmacological properties in patients suggest that fosaprepitant has the potential to generate a measurable response in combination with current treatment regimens in patients with high-risk neuroblastoma, and thus, should be considered for entry into clinical testing.

## MATERIALS AND METHODS

### Reagents and cell culture

All reagents were obtained from Sigma Aldrich if not otherwise specified (Sigma Aldrich, St. Louis, MO, USA). The identity of the human neuroblastoma cell lines, IMR5, SK-N-BE, SK-N-AS, SY5Y and Kelly, and foreskin fibroblasts were verified by STR genotyping performed by the German Collection of Microorganisms and Cell Cultures (DSMZ, Braunschweig, Germany). Cell lines were cultured at 37°C in a humidified atmosphere, with 5% CO_2_ in RPMI 1640 supplemented with 10% fetal calf serum, 1% L-glutamine, and 100 U penicillin/streptomycin per ml medium. Substance P and acetate salt (Sigma-Aldrich) were dissolved in distilled water to 10 mM (stock solution) and diluted to 5, 10, 50, 100 and 500 nM in full medium for experiments. In experiments investigating synergistic or competing effects, the IMR5 cell line was incubated 1 h with substance P before adding other treatments. Fosaprepitant (IVEMEND) was dissolved in 0.9% NaCl for a 10 mM stock solution that was aliquoted for single use in both cell culture and mouse experiments and stored at 4°C. Meglumine (dissolved in 0.9% NaCL to 10 mM and stored at 4°C) was used for control cell cultures and mice, since it is the most abundant substance in fosaprepitant powder after the active agent.

### Viability, proliferation and cell death quantification

Cell lines were seeded onto 96-well plates (2 × 10^3^ per well) in at least triplicate for all assays, and incubated for 24 h to permit surface adherence. Concentrations of 1–100 μM fosaprepitant were prepared by serial dilution in complete medium, and cell viability was assessed in time course after 24 h, 48 h, 72 h, 96 h and 120 h of treatment using the Cell proliferation, MTT assay (Roche, Basel, Switzerland), according to the manufacturer's protocol. Absorbance was read at 570 nm on an AD340 plate reader. Growth inhibition dose-response curves were plotted as a percentage of untreated control cells. Fifty percent inhibition of growth (IC50) and 95% confidence interval (CI) were calculated using GraphPad Prism 5.0 (GraphPad Software Inc., San Diego, CA, USA). Apoptosis and proliferation were assessed after 48 h and 72 h of treatment with 25 μM fosaprepitant using the Cell Death Detection ELISA and Cell proliferation BrdU ELISA (Roche) assays (Roche, Basel, Switzerland) according to manufacturer's instructions. For FACS-based cell cycle analyses, cell lines were cultured for 24–72 h with 10 μM or 25 μM fosaprepitant or 425 μM meglumine as a control in 22 mm plates initially seeded with 1 × 105 cells/plate. Cells were trypsinized, washed 3 times with phosphate-buffered saline, then incubated with DAPI and Annexin V (ANXA5) as previously described [26] before analysis on a FC500 flow cytometer (Beckman Coulter). All experiments were independently performed 3 times, unless otherwise indicated. Experiments to assess substance P competition of fosaprepitant treatment were conducted using IMR5 cells seeded into 96-well plates at 3000 cells/well. Substance P was added 24 h after seeding, and fosaprepitant was added 1 h later, then cell viability was measured after 72 h of treatment using the MTT assay, as described above. To assess cellular survival in real-time using the Xcelligence system (Roche, Basel, Switzerland), cells were plated in triplicate at 2 × 103 cells/well onto 96-well Xcelligence microelectronic cell sensor plates, and cultured overnight in antibiotic-free complete media. Cells were treated with Fosaprepitant (5 μM, 10 μM or 25 μM) or Meglumine vehicle control, then adherence to the culture plates was continuously monitored for 190h to assess cellular survival. For cell cycle analysis, cell lines were cultured 48h with Fosaprepitant (10 μM or 25 μM) or Meglumine control in 35mm plates at 5 × 107 cells/plates. Cells were removed by trypsinization and washed 3 times with PBS, then incubated with propidium iodide for 15 min to stain DNA. Cellular DNA content was analyzed in an FC500 flow cytometer (Beckman Coulter). All experiments were independently performed at least 3 times, if not otherwise indicated.

### Western blot analysis

Protein lysate preparation and western blotting was carried out as described previously (13) using primary antibodies against TACR1 (1:500, NB300-119, Novus biologicals, R&D Systems GmbH, Wiesbaden, Germany), SRC (1:500, #2108, Cell signaling technologies, Danvers, MA, USA), p-SRC (1:500, #2101, Cell signaling technologies), Actin (1:5000, #3700, Cell signaling technologies), AURKB (1:2000, ab45145, Abcam, Germany) and GAPDH (1:2000, MAB374, Millipore). After washing twice with 0.1% Tween 20 in Tris-buffered saline, pH = 7.5 (TBS-T), membranes were incubated 1h at room temperature with either horseradish peroxidase-conjugated anti-mouse IgG (#NA9310V; GE Healthcare, Solingen, Germany), anti-rabbit IgG (#NA9340V; GE Healthcare) or anti-sheep IgG (#HAF016; R&D Systems), diluted 1:2000 in 5% nonfat dry milk in TBS-T. Proteins were visualized using the ECLplus western blotting detection kit (GE Healthcare) and analyzed on the FusionFX7 detection device (Peqlab, Erlangen, Germany). Densitometry analysis was performed using ImageJ according to the programs recommendations (https://imagej.nih.gov).

### Quantitative RT-PCR analysis

Quantitative RT-PCR analysis was done as previously described [27]. In short, total RNA was isolated from cells using the RNeasyMini kit (Qiagen, Hilden, Germany), and cDNA synthesis was performed using the SuperScript reverse transcription kit according to the manufacture's protocol (Invitrogen, Darmstadt, Germany). *TACR1* expression were monitored using Assays-on-Demand™ (Applied Biosystems, Foster city,CA, USA). Expression values were normalized to the geometric mean of *GAPDH* [28]. Data analysis and error propagation were performed using the qbase^PLUS^ software version 1.5 (http://www.biogazelle.com).

### Microarray expression analysis

IMR5 cells were plated at 2 × 10^5^ cells/well in 6-well plates, left for 12 h to attach, then treated in triplicate with medium containing 425 μM meglumine (control) or 10 μM or 25 μM fosaprepitant for 24 h. Total RNA was extracted using the RNeasyMini kit (Qiagen, Hilden, Germany), and samples profiled on the HG-U133 Plus 2.0 Human Gene Expression Array (Affymetrix, Santa Clara, CA, USA) at the Centre for Medical Biotechnology, University Hospital Essen using established protocols.

### Data analysis

For microarray based gene-expression analysis, microarray CEL files were normalized and summarized over probes for gene expression using the Bioconductor gcRMA normalization tool in R [29]. Probes for which log2 expression was < 4 in 7 of 8 samples, were considered underexpressed and not used in analyses, leaving a total of 11568 unique genes for analysis. Ward-Manhattan clustering of the 300 probes with the highest standard deviation over all selected samples was performed to evaluate the similarity between samples. Differential expression was analyzed using Rank-Product analysis in R (v 2.13, RankProd package) [30]. Hierarchical clustering on Manhattan distance of log2 expression values of the 50 most differentially expressed genes was applied to visualize differential gene expression after treatment. Regression analysis with FDR correction for multiple testing was used to identify genes increasing or decreasing in expression over the applied concentration range, and genes with a log2 fold-change of at least 1 between the highest treatment concentration and control condition were considered as differentially expressed. In practice, this was detected by a regression line for a gene with a slope > 0.4 or < −0.4, which indicated gene up- or downregulated, respectively. To gain insight into transcriptional programs downstream of TACR1 receptor signalling, iRegulon analysis, a Cytoscape plugin to identify regulons in a set of co-regulated genes using motif discovery, was performed on up- and downregulated genes [31]. A regulon consists of a transcription factor and its direct transcriptional targets, which contain common transcription factor binding sites in their cis-regulatory control elements. Gene set enrichment analysis was performed using the c2.cgp.v3.1.symbols.gmt gene set and GSEA v2.0 software from www.broadinstitute.org/gsea [32]. Genes were rank-ordered using a signal-to-noise ranking metric that scales the difference of means in the populations to be compared using the standard deviation.

### Fosaprepitant treatment of neuroblastoma xenograft tumors in nude mice

IMR5 cells were cultured to 80% confluence, harvested and suspended in 200 μl Matrigel™ (BD Bioscience, Heidelberg, Germany) for subcutaneous inoculation (2 × 107 cells per mouse) into the left flank of 6-week-old female athymic (nu/nu) mice. Mice were randomly assigned to vehicle control or fosaprepitant groups (*n* = 7 mice per group) after tumors had clearly progressed and reached 150 200 mm3 in size. Fosaprepitant was administered by intraperitoneal injection at 60 mg/kg body weight (BW) daily. Vehicle controls were treated with meglumine in 0.9% NaCl (60 mg/kg BW daily). Tumor growth was monitored using calipers and tumor volume was calculated using the formula (width × length × height)/2. Mice were sacrificed by cervical dislocation after 15 days of treatment. All animal experiments were performed in accordance with the Council of Europe guidelines for housing and care of laboratory animals, and protocols were approved by the Ethical Commission for Animal Experimentation at the University Hospital Essen.
